# Immunogenetic aspects of idiopathic recurrent miscarriage in the Kazakh population

**DOI:** 10.25122/jml-2021-0063

**Published:** 2021

**Authors:** Gulnara Svyatova, Dinara Mirzakhmetova, Galina Berezina, Alexandra Murtazaliyeva

**Affiliations:** 1. Republican Medical Genetic Consultation, JSC Scientific Center of Obstetrics, Gynecology and Perinatology, Almaty, Republic of Kazakhstan

**Keywords:** gene polymorphism, genotypes, histocompatibility complex, implantation, pregnancy, reproductive medicine

## Abstract

There are numerous scientific studies of recurrent miscarriage (RM) with possible causes, such as fetal chromosomal abnormalities, infectious agents, adverse environmental factors, bad habits, anatomical defects, thrombophilic disorders, etc. However, RM causes in 50% of cases remain unknown. These RM cases do not have any explainable etiology, and they require in-depth etiopathogenesis study, thus they are considered idiopathic RM. The purpose of this research is to study polymorphisms relationship of the immune response genes CX3CR1 (rs3732379, Val249Ile), CTLA4 (rs3087243, CT60 G/A), and HLA DQA1, DQB1, DRB1 (major histocompatibility complex, class II) with the idiopathic form of recurrent miscarriage (iRM) development in Kazakh population. Independent replicative TagMan genotyping for 302 patients with iRM and 300 women with normal reproduction was performed. It has been shown that carriage of unfavorable genotypes (Val/Ile, Val/Val) by the Val249Ile polymorphism of the CX3CR1 gene increases the risk of developing iRM by 1.43 times. Search for associations of genes allelic variants of HLA class 2 complex with iRM revealed *501 allele in DQA1 locus, *0301 in DQB1 locus, *10, *12, *15, *16 alleles in DRB1 locus, which increases the risk of developing iRM in Kazakh population with OR from 1.34 to 4.5. As a result of the study, obtained highly significant associations of immune response genes with the development of iRM in the Kazakh population indicate the possible involvement of the immune system interaction of mother cells with syncytiotrophoblast, which is realized by vascularization defects, defective embryo implantation, and leads to early pregnancies’ termination.

## Introduction

Recurrent miscarriage (RM) is a heterogeneous disorder (two or more spontaneous miscarriages) in the reproductive period that affects up to 3% of couples [1–3]. The American Society for Reproductive Medicine (ASRM) considers that the recurrence rate and risk factors for two consecutive pregnancy losses are similar to those observed after three losses [1, 4–11]. The independent replicative genotyping of Genome-Wide Association Studies (GWAS) associated with RM polymorphisms in the Kazakh population did not confirm the genetic contribution of coagulation and cardiovascular system, anti-inflammatory cytokines, apoptosis, and angiogenesis genes polymorphism in iRM development [12–14]. Many authors [4, 5, 7–9, 15] point to a significant role in violations of mother and fetus immune tolerance mechanisms in iRM, where the main protective role in the early stages of pregnancy is played by regulatory T cells of the mother’s immune system. Cytotoxic T-Lymphocyte Associated Protein 4 (CTLA4) is a key immunoregulatory molecule that is involved in the suppression of T cell activation [10, 15]. CTLA4 is one of the co-stimulatory molecules CTLA4 is expressed on CD4+CD25+ regulatory T cells and is responsible for their activation and proliferation. It has been shown that decreased expression of CTLA4 in the placenta can weaken the inhibition of activated T-lymphocytes, impair immunity, which leads to RM development [10]. Several studies have shown that CTLA4 gene rs3087243 mutant genotypes contributed to CTLA4 level decrease in the blood serum and led to RM [15–18]. It is known that fractalkine regulates the early stages of pregnancy, promotes blastocyst implantation, participates in the remodeling of uteroplacental arteries. During the first week of pregnancy, immunohistochemical analysis of endometrium showed maximum expression of fractalkine in uterus glandular epithelium; this activates chemokine receptors and contributes to successful blastocyst implantation [19, 20]. Mutant genotypes carriage reduces production, binding fractalkine to CX3CR1 receptor, leading to adhesion and migration disruption of fetal and maternal cells, causing spontaneous abortion [19–21].

HLA DQA1, DQB1, DRB1 genes – classic human leukocyte antigen, major histocompatibility complex class II. HLA DQA1, DQB1, DRB1 genes are included in the major histocompatibility complex, which encodes many immunological proteins, including classic human leukocyte antigen (HLA). In recent years, the role of HLA has been widely studied in the genesis of recurrent miscarriage, which is represented by more than 150 antigens [22–26]. Trophoblast antigenic composition is mainly represented by antigens histocompatibility in the II class, which allows them to be used as immunological markers of RM development [27]. There are publications that HLA complex suppresses the maternal immune response required for implantation. It is assumed that in the presence of a homogeneous HLA histocompatibility complex (common for the mother and father) in inbred populations, the frequency of RM increases [28, 29]. The choice of polymorphisms CTLA4 (rs3087243, CT60 G/A), CX3CR1 (rs3732379, Val249Ile), genes HLA DQA1, DQB1, DRB1 (class II major histocompatibility complex) for the analysis of immune processes in iRM is statistically determined by their participation in T-cell tolerance, suppressing significant associations with RM according to GWAS data [2, 4, 5, 8, 9]. There are numerous scientific studies of RM possible causes, such as infectious agents, adverse anatomical defects, environmental factors, thrombophilic disorders, etc. However, RM causes in 50% of cases remain unknown [2–6]. These RM cases do not have any explainable etiology, and require in-depth etiopathogenesis study, thus are considered idiopathic RM. Evidence for a multifactorial genetic etiology of iRM will increase by eliminating known clinical, laboratory, and environmental risk factors. The absence of large-scale GWAS studies devoted to iRM is due to several objective reasons: lack of clear iRM definitions, difficulty recruiting and small sample size, and insufficient replicative studies in ethnically similar populations [7–11]. The purpose of the research is to assess the possible contribution of immune response genes for the development of iRM, such as CTLA4 (rs3087243, CT60 G/A), CX3CR1 (rs3732379, Val249Ile), HLA DQA1, DQB1, DRB1 in the Kazakh population.

## Material and Methods

The authors used published GWAS meta-analyses results for “candidate genes” of RM involved in the immune response, coagulation, metabolism, angiogenesis, placental function, and chromosomal segregation [8, 9, 12]. The study was carried out by a prospective method in the outpatient department of the Scientific Center for Obstetrics, Gynecology, and Perinatology (SCOPG), Medical Center “Molecular Medicine Center”. The study group with iRM consisted of 302 women of Kazakh; aged 18–45; who had had two or more miscarriages before 12 weeks of pregnancy. The control group is represented by 300 female Kazakh women with normal reproductive function, with at least one child, without any indication of spontaneous miscarriages.

Exclusion criteria: luteal phase abnormalities in the results of endometrial biopsy, anatomical abnormalities of the uterus diagnosed by hysterosalpingography, hysteroscopy or sonohysteroscopy, carriage of balanced chromosomal abnormalities by karyotyping of both spouses, the presence of antiphospholipid syndrome, confirmed by anti-beta I2-glycoprotein analysis anti-cardiolipin (IgG or IgM) antibodies, lupus anticoagulant; multiple pregnancies, confirmed by ultrasound, the presence of sexually transmitted infections, confirmed by two different analyzes of various biological materials (enzyme-linked immunosorbent assay, PCR), thyroid dysfunction according to TSH and thyroid antibodies. DNA isolation was performed by separating magnetic particles M-PVA on automatic analyzer Prepitto (PerkinElmer) to isolate nucleic acids ChemagicPrepito (Wallac, Finland) using the PrepitoDNACytoPure reagent kit. Molecular genetic studies were carried out by the TaqMan method of unified site-specific amplification and real-time genotyping (Real-Time PCR) using test systems (TestGen, Russia) for molecular genetic studies. Statistical significance tests and χ2 analysis were performed using PLINK, STATA13 software. Differences in allelic and genotypic frequencies were assessed using the χ2 test with odds ratio (OR) [30].

## Results

As shown in Table 1, replicative studies in the Kazakh population revealed a significant association of alleles and genotypes (rs3087243) of CTLA4 gene polymorphism with iRM risk developing (p>0.05). The frequency of allele A was relatively high in comparison groups: study group – 29.47%, control group – 33.83% (χ2=2.65; p>0.05). Noteworthy is the low frequency of the mutant AA genotype in the study group – 6.95%, the differences were not statistically significant (χ2=3.47; p=0.06). Similar results were obtained in most of the conducted replicative studies of rs3087243 CTLA-4 gene polymorphism with iRM in women from northwestern Iran [10], northern India [11], China [31, 32]. Only one large study conducted by Wang *et al.* in the Chinese population revealed a significant contribution of the studied polymorphism to the immunopathogenesis of iRM [33]. As shown in Table 1, we obtained statistically significant differences in frequency alleles and genotypes of Val249Ile fractalkine receptor 1 CX3CR1 polymorphism in the compared groups (p<0.05). The frequency of the minor Val allele in the iRM group was significantly higher, amounted to 22.02% (χ2=5.89; p=0.02). Accordingly, wild homozygous genotype Ile/Ile was found in the group of patients with iRM – 59.93%, which was significantly lower than in the control group – 69.93% (p=0.02). Significant differences were not found in unfavorable homozygous Val/Val genotype frequency (χ2=1.33; p=0.25), probably due to its low frequency in both compared groups – 3.97 and 2.33, respectively.

**Table 1.** Frequency alleles and genotypes of immune response genes in the group with iRM and the control group.

**Gene/polymorphism**	**Genotypes/alleles**	**Study group** **(n=302) n (%)**	**Control group** **(N=300) n (%)**	**χ^2^**	**p**
**CTLA4 (CT60 G/A)**	GG	145 (48.0%)	131 (43.7%)	1.15	0.285
	GA	136 (45.0%)	135 (45.0%)	0.0	0.994
	AA	21 (7.0%)	34 (11.3%)	3.47	0.063
	G	426 (70.5%)	397 (66.2%)		
	A	178 (29.5%)	203 (33.8%)	2.65	0.104
**CX3CR1 (Val249Ile)**	Ile/Ile	181 (60.0%)	208 (69.3%)	5.82 *	0.016
	Val/Ile	109(36.1%)	85 (28.3%)	4.15 *	0.042
	Val/Val	12 (3.9%)	7 (2.4%)	1.33	0.250
	Ile	471 (78.0%)	501 (83.5%)		
	Val	133 (22.0%)	99 (16.5%)	5.89 *	0.016

* identified significant differences p<0.05.

We found only one population-based retrospective study in the scientific literature, conducted in North India, which revealed highly significant associations between CX3CR1 gene polymorphism (rs3732379) with iRM [34]. The authors obtained significant differences with a high frequency of unfavorable genotypes CX3CR1 rs3732379 gene carriage in iRM group. It may indicate the involvement of the immune response system in the process of implantation through an insufficient expression of the CX3CR1 receptor in invasive trophoblast cells. This, in turn, disrupts the immune processes of maternal cells’ interaction with syncytiotrophoblast and leads to early termination of pregnancy. As shown in Table 1, carriage of unfavorable genotypes (Val/Ile, Val/Val) for the Val249Ile CX3CR1 gene polymorphism increases the risk of developing iRM by 1.43 times (OR=1.43; 95% CI=1.03–2.02). Statistical analysis using PLINK includes the calculation of associations based on various models. The results of tests performed on the authors’ sample data, including genotypic (GENO), additive (TREND), allelic (ALLELIC), and dominant (DOM) models, revealed significant associations with iRM, which imply a specific relationship between genotype and phenotype, for the CX3CR1 gene immune response (rs3732379, Val249Ile) (χ2=6.15, 6.11, 5.89 and 5.82; p<0.00001, according to the models) [30]. Table 2 shows the results of the possible contribution analysis for the multi-allelic loci HLA DQA1, DQB1, DRB1 – major histocompatibility complex class II to the development of iRM in the Kazakh population.

**Table 2.** Allelic frequencies of HLA DQA1, DQB1, and DRB1 genes in the study and control groups.

**Gene/polymorphism**	**Genotypes/alleles**	**Study group** **n (%)**	**Control group** **n (%)**	**χ^2^**	**p**	**OR** **DI 95%**
**HLA DQA1**	0101	92 (15.2%)	90 (15.0%)	0.01	0.911	1.02 (0.74–1.39)
	0102	80 (13.3%)	87 (14.5%)	0.39	0.529	0.90 (0.65–1.25)
	0103	60 (9.9%)	75 (12.5%)	1.99	0.159	0.77 (0.54–1.11)
	0201	87 (14.4%)	97 (16.2%)	0.72	0.396	0.87 (0.64–1.20)
	0301	87 (14.4%)	81 (13.5%)	0.21	0.651	1.08 (0.78–1.49)
	0401	20 (3.3%)	22 (3.7%)	0.11	0.737	0.90 (0.49–1.67)
	0501	166 (27.5%)	132 (22.0%)	4.86 *	0.028	1.34 (1.03–1.75)
	0601	12 (2.0%)	16 (2.6%)	0.61	0.434	0.74 (0.35–1.58)
**HLA DQB1**	02	123 (20.4%)	120 (20.0%)	0.03	0.875	1.02 (0.77–1.36)
	03	8 (1.3%)	22 (3.7%)	6.79 *	0.010	2.90 (0.16–0.80)
	0301	135 (22.4%)	103 (17.2%)	5.10 *	0.024	1.39 (1.04–1.85)
	0302	40 (6.6%)	54 (9.0%)	2.36	0.125	0.72 (0.47–1.10)
	0303	28 (4.6%)	29 (4.8%)	0.03	0.872	0.96 (0.56–1.63)
	0304	4 (0.7%)	2 (0.3%)	0.66	0.418	1.99 (0.36–10.9)
	0305	3 (0.5%)	3 (0.5%)	0.000	0.994	0.99 (0.20–4.94)
	0401/0402	31 (5.1%)	32 (5.3%)	0.02	0.876	0.96 (0.58–1.59)
	05	11 (1.8%)	14 (2.3%)	0.39	0.534	0.78 (0.35–1.72)
	0501	47 (7.8%)	48 (8.0%)	0.02	0.889	0.97 (0.64–1.48)
	0502/0504	27 (4.5%)	17 (2.8%)	2.29	0.131	1.61 (0.87–2.98)
	0503	19 (3.2%)	25 (4.2%)	0.89	0.346	0.75 (0.41–1.37)
	0601	24 (3.9%)	19 (3.2%)	0.57	0.451	1.27 (0.69–2.34)
	0602-8	104 (17.2%)	112 (18.7%)	0.43	0.513	0.91 (0.68–1.22)
**HLA DRB1**	01	29 (4.8%)	43 (7.2%)	3.00	0.840	0.65 (0.40–1.06)
	03	57 (9.4%)	51 (8.5%)	0.32	0.570	1.12 (0.76–1.67)
	04	87 (14.4%)	94 (15.7%)	0.38	0.540	0.91 (0.66–1.24)
	07	86 (14.2%)	84 (14.0%)	0.01	0.906	1.02 (0.74–1.41)
	08	32 (5.3%)	35 (5.8%)	0.16	0.686	0.90 (0.55–1.48)
	09	31 (5.1%)	34 (5.7%)	0.17	0.682	0.90 (0.55–1.49)
	10	15 (2.5%)	5 (0.8%)	5.02 *	0.026	3.03 (1.09–8.39)
	11	55 (9.1%)	53 (8.8%)	0.03	0.869	1.03 (0.69–1.54)
	12	37 (6.1%)	21 (3.5%)	4.53 *	0.034	1.80 (1.04–3.11)
	13	65 (10.8%)	86 (14.3%)	3.50	0.062	0.72 (0.51–1.02)
	14	38 (6.3%)	51 (8.5%)	2.15	0.144	0.72 (0.47–1.12)
	15	63 (10.5%)	41 (6.9%)	4.94 *	0.027	1.59 (1.05–2.39)
	16	9 (1.5%)	2 (0.3%)	4.45 *	0.035	4.52 (0.97–21.2)

* identified significant differences p<0.05.

Pairwise comparative analysis of allelic variants frequency in the HLA DQA1 gene revealed a significant excess of 0501* allele carriage in the study group – 27.48%, in the control group – 22.00% (χ2=4.86; p=0.03). There is a significant association of the HLA DQA1 gene 0501* allele with the iRM developing risk in the Kazakh population (OR=1.34; 95% CI=1.03–1.75.) Table 2 shows that the most frequent HLA DQB1 gene allelic variant in iRM group was allele 0301*, which was 22.35% and significantly exceeded the same indicator in the control group – 17.17% (χ2=5.10; p=0.02). As shown by calculations, the carriage of allele 0301* increases the risk of iRM developing in the Kazakh population by 1.4 times (OR=1.43; 95% CI=1.04–1.85). The frequency of protective HLA DQB1 gene allele 03* in the study group was 1.32%, which turned out to be significantly lower in the group of women with normal reproduction – 3.67% (χ2=6.80; p=0.01). Carriage of protective allele 03* of the main histocompatibility complex class II – HLA DQB1 gene reduces the risk of iRM developing by 2.9 times (OR=2.90; 95% CI=1.27–6.64) (Table 2). In the HLA DRB1 gene, the authors found four allelic variants reliably associated with iRM – 10*, 12*, 15*, 16*. Frequency carriage in the study group was significantly higher than similar values in the control group (p<0.05). Carriage of allele 10* increases the risk of iRM developing by 3 times, allele 12* – 1.8 times, allele 15* – 1.6 times. The highest risk with the rare 16* allele was found, which increases the risk of iRM developing by 4.5 times.

## Discussion

Scientific literature discusses the role of homozygous genotypes carriage for the risk alleles of the main complex of tissue compatibility HLA class II – HLA DQA1, DQB1, DRB1 genes in the development of RM, which was confirmed by studies of several other diseases [35, 36]. In one study, the occurrence of homozygotes was significantly higher for the HLA DRB1*04 allele frequency in women with iRM, which suggests the contribution of homozygosity for risk alleles to the development of reproductive losses [37]. The frequency was determined for homozygous genotypes *0501/0501 HLA DQA1 gene, study group – 10.92%, control group – 6.67%, but the differences are not statistically significant (p>0.05). Homozygous genotypes for the rare alleles *10, *12, *16 of the HLA DRB1 gene were low in study and control groups. Significant differences were found for the homozygous genotype of the HLA DQB1*0301/0301 gene – 7.28% in women with iRM, in the control group – 3.00% (χ2=5.66; p=0.018). The results obtained suggest an increased iRM developing with the carriage of two risk alleles. The complexity of the immunological relationship between mother and fetus during pregnancy dictates the need to continue research, study additional SNP genes of the immune response. The results obtained by I. Tkach [38] during genotyping of Ukrainian women with RM – HLA DQB1*0301 and DQA1*0501 are similar to risk alleles for the Kazakh population. A. Iskhakov [39] showed a significant HLA DRB1 gene 15* allele contribution to the increased risk of RM development. It should be noted that in several large studies [37, 40], alleles and genotypes were identified, which did not coincide with the authors’ results.

## Conclusions

This work confirms the need to conduct independent replicative studies in each population since genetic polymorphisms are associated with the geographical, ethnic, and historical characteristics of each group. It is impossible to extrapolate the highly significant associations of even large-scale GWAS analyzes and use them as genetic markers for iRM without replicative genotyping, as this would lead to false-positive results. The independent replicative genotyping GWAS excluded the genetic contribution of coagulation and cardiovascular system, anti-inflammatory cytokines, apoptosis, and angiogenesis genes for iRM in the Kazakh population. For the first time it was made possible to determine the etiological conditionality of idiopathic forms of RM by disorders of the immune interaction of maternal cells with syncytiotrophoblast, and the carriage of HLA class II complex risk alleles.

The obtained highly significant associations for various models of the CX3CR1 gene (rs3732379, Val249Ile) increase the risk of developing iRM with OR 1.4 times. This indicates the possible involvement of the immune response system, which is realized by vascularization defects, inadequate embryo implantation and leads to early termination of pregnancy. An independent replicative study to search for associations of allelic variants of HLA class II complex genes with iRM revealed an increased risk in the Kazakh population from OR=1.34 to 4.5. We found protective allele HLA DQB1*03; its carriage reduces the risk of iRM by 2.9 times (OR=2.90; 95% CI=1.27–6.64).

## Acknowledgements

### Conflict of interest

The authors declare that there is no conflict of interest.

### Ethics approval

The study was approved by the National Ethics Commission of the Ministry of Health of the Republic of Kazakhstan on November 15, 2020, No 1721-A. All procedures performed in studies involving human participants were following the ethical standards of the institutional and national research committee and the 1964 Helsinki declaration and its later amendments or comparable ethical standards.

### Consent to participate

All study participants gave informed consent to use their blood samples and collect anamnestic data; permission was obtained from the ethical committee of the SCOPG to conduct studies.

### Funding

The study received funding from the framework of 49019\ПЦФ-M3CP-OT-18.

### Authorship

GS, and DM took part in conceptualization, project administration, formal analysis, writing – review and editing. GB and AM performed data curation, formal analysis, methodology, and writing – review and editing.
